# We’re only in it for the knowledge? A problem solving turn in environment and health expert elicitation

**DOI:** 10.1186/1476-069X-11-S1-S3

**Published:** 2012-06-28

**Authors:** Hans Keune, Arno C Gutleb, Karin E Zimmer, Solveig Ravnum, Aileen Yang, Alena Bartonova, Martin Krayer von Krauss, Erik Ropstad, Gunnar S Eriksen, Margaret Saunders, Brooke Magnanti, Bertil Forsberg

**Affiliations:** 1 Research Institute for Nature and Forest (INBO), Brussels; Centre of Expertise for Environment and Health, Faculty of Political and Social Sciences, University of Antwerp; naXys, Namur Center for Complex Systems, University of Namur, Belgium; 2 Department of Environment and Agro-biotechnologies (EVA), Centre de Recherche Public - Gabriel Lippmann, Luxembourg; 3 Department of Basic Sciences and Aquatic Medicine, Norwegian School of Veterinary Science, Norway; 4 NILU - Norwegian Institute for Air Research, Norway; 5 Norwegian Veterinary Institute, Norway; 6 WHO, Regional Office for Europe, Denmark; 7 Department of Production Animal Clinical Science, Norwegian School of Veterinary Science, Norway; 8 University Hospitals Bristol NHS Foundation Trust, England; 9 Occupational and Environmental Medicine, Umea University, Sweden

## Abstract

**Background:**

The FP6 EU HENVINET project aimed at synthesizing the scientific information available on a number of topics of high relevance to policy makers in environment and health. The goal of the current paper is to reflect on the methodology that was used in the project, in view of exploring the usefulness of this and similar methodologies to the policy process. The topics investigated included health impacts of the brominated flame retardants decabrominated diphenylether (decaBDE) and hexabromocyclododecane (HBCD), phthalates highlighting di(2-ethylhexyl)phthalate (DEHP), the pesticide chlorpyrifos (CPF), nanoparticles, the impacts of climate change on asthma and other respiratory disorders, and the influence of environment health stressors on cancer induction.

**Methods:**

Initially the focus was on identifying knowledge gaps in the state of the art in scientific knowledge. Literature reviews covered all elements that compose the causal chain of the different environmental health issues from emissions to exposures, to effects and to health impacts. Through expert elicitation, knowledge gaps were highlighted by assessing expert confidence using calibrated confidence scales. During this work a complementary focus to that on knowledge gaps was developed through interdisciplinary reflections. By extending the scope of the endeavour from only a scientific perspective, to also include the more problem solving oriented policy perspective, the question of which kind of policy action experts consider justifiable was addressed. This was addressed by means of a questionnaire. In an expert workshop the results of both questionnaires were discussed as a basis for policy briefs.

**Results:**

The expert elicitation, the application of the calibrated confidence levels and the problem solving approach were all experienced as being quite challenging for the experts involved, as these approaches did not easily relate to mainstream environment and health scientific practices. Even so, most experts were quite positive about it. In particular, the opportunity to widen one’s own horizon and to interactively exchange knowledge and debate with a diversity of experts seemed to be well appreciated in this approach. Different parts of the approach also helped in focussing on specific relevant aspects of scientific knowledge, and as such can be considered of reflective value.

**Conclusions:**

The approach developed by HENVINET was part of a practice of learning by doing and of interdisciplinary cooperation and negotiation. Ambitions were challenged by unforeseen complexities and difference of opinion and as no Holy Grail approach was at hand to copy or follow, it was quite an interesting but also complicated endeavour. Perfection, if this could be defined, seemed out of reach all the time. Nevertheless, many involved were quite positive about it. It seems that many felt that it fitted some important needs in current science when addressing the needs of policy making on such important issues, without anyone really having a clue on how to actually do this. Challenging questions remain on the quality of such approach and its product. Practice tells us that there probably is no best method and that the best we can do is dependent on contextual negotiation and learning from experiences that we think are relevant.

## Background

### The ambition of knowledge evaluation

To protect the health of populations and individuals, policies need to integrate environmental and health issues. The ambition of the FP6 EU HENVINET project was to support such informed policy making. HENVINET was expected to review, exploit and disseminate knowledge on environmental health issues based on research and practices, for wider use by relevant stakeholders. Furthermore, it was expected to lead to validation of tools and results with emphasis on the four priority health endpoints (cancer, asthma and allergy, neurodevelopment and endocrine disruption) of the European Environment and Health Strategy (EHAP) 2004-2010, and to provide structured information overview that may be utilized by other actors relevant to the EHAP [1]. Building on previous EU funded activities such as AirNET, CLEAR, PINCHE, INTARESE and SCALE, HENVINET had planned to collect, structure and evaluate new material and present it in a consistent manner, which would lend itself to transparency and identification of knowledge gaps. Given the goals of the HENVINET project and the EHAP, two ambitions regarding the nature and purpose of the knowledge were considered to be most relevant:

1. The knowledge should pertain directly to the causal relationships between environmental health stressors and the impacts on health.

2. The knowledge will be used in a policy context, rather than in a research context.

### Expert elicitation

The HENVINET project used the expert elicitation method to highlight knowledge gaps in the four chosen environmental health topics. When using this method, experts in the different topics are consulted for advice. One of the first formal expert elicitation methods was the Delphi method [2,3], and many other studies have been published e.g. from the IPCC [4], European Environmental Agency [5] and U.S. Environmental Protection Agency [6]. The method has been criticized [7,8], but it is still one of the best options to support policy making when knowledge is considered limited.

Expert elicitation is one of the umbrella terms under which a variety of methods for involving experts in knowledge assessment can be found. As Knol et al. [7] describe it, it is *“a structured approach of consulting experts on a subject where there is insufficient knowledge and seeks to make explicit the published and unpublished knowledge and wisdom of experts. Expert elicitation can serve as a means to synthesize the* (*limited*) *available knowledge in order to inform policies which have to be made before conclusive scientific evidence becomes available”.* As such, expert elicitation relates well to the challenge of complexity: situations where scientific knowledge is limited, facts are uncertain, the stakes are high and values are conflicting [9]. In dealing with complexity in the field of environment and health in general, two strategies may be considered: a problem knowledge oriented strategy and a problem solving oriented strategy [10,11]. Both strategies can be combined in a complementary manner, as is the ambition of the analytical deliberative approach proposed by Stern and Fineberg [12] and the extended peer review proposed by Pereira and Funtowicz [13], in which expert perspectives are combined with social perspectives. A key issue is the balancing of problem knowledge and problem solving: when do we know enough about public health risks in order to implement policy measures? This is especially important when one considers the issues at stake to be not only socially important, but also by definition characterized by limited knowledge because of complexity [10,14].

An important issue of concern for expert elicitation is the issue of quality: how can we promote and judge the quality of an expert elicitation? Knol et al. [7] mainly approach this from a problem knowledge perspective, as they mainly focus on knowledge uncertainties. Krayer von Krauss [10] addresses this issue by pointing out that the relationship between uncertainties and quality is not straightforward: *“Quality can be defined as “the totality of characteristics of an object that bear on its ability to satisfy an established need”. Whereas uncertainty is an attribute of knowledge*, *the quality of knowledge is an attribute of the relationship between knowledge and the purpose for which it is intended to be used. Thus*, *depending on the function for which it is intended*, *uncertain knowledge may still be considered of good quality” and “There can be no absolute definition of good or bad quality*, *and it is only possible to arrive at quality judgments through collective reflection and deliberation on the information available*, *in view of the policy context in which it is to be used.”*

In this paper we describe how expert elicitation was used in practice as a means for knowledge evaluation and policy interpretation. We describe experiences from several cases.

## Methods

We present the general methodology that was applied in practice and in fact was part of practice: it developed during a complicated but interesting interdisciplinary endeavour of learning by doing, negotiation and trial and error. The method therefore is to be considered as part of the process and as such as a result of the project. Nevertheless we find it more relevant to present the methodology here, and to present the experiences and evaluation of practice from different actors involved in the project in the results section.

### First phase: knowledge evaluation

The initial understanding of the topic experts within the 4 topics was that at least one review paper on a chosen compound or of a factor related to their area of expertise would be the output of the project. Systematic reviews, contrary to traditional opinion based narrative reviews, attempt to minimize bias by a comprehensive and reproducible selection of articles to base the review upon. Therefore the systematic approach to produce the reviews was chosen, which implies the development of a search strategy, search phrases and words, and inclusion and exclusion criteria before the actual literature search starts [15]. However, as an example, this resulted in >1000 articles for phthalates that would go far beyond a workable amount. At this stage it was decided to use available reviews from the National Toxicology Program [16] and European Food Safety Authority [17,18] as “golden standards” followed by a search in PubMed only for DEHP as an important representative for phthalates including only papers published in 2005 or later, which resulted in 191 references (search performed 19.09.2007).

O’Malley et al. [19] pointed out that the quality of environmental information with which one claims to influence policy discussions, must ‘go beyond the basic notions of scientific excellence’. ‘For information to be used and useful, and not itself be the subject of debate, it should meet three standards: it should be policy relevant, technically credible and politically legitimate’. Jasanoff [20] warns against the danger of politicians using expertise that serves specific political agendas rather than expertise that may yield robust findings that could be unacceptable to the political agenda. It was agreed that in addition to the type of quality parameters conventionally applied in research science, measurements of the quality of policy relevant science must also take account of the extent to which the various actors in a given policy process accept the knowledge as the legitimate frame of reference for policy making. It was decided that a set of knowledge assessment criteria that aim to account for the policy context within which the knowledge in question would be used. Two sets of criteria were proposed, the first for the assessment of empirical information and the second for the assessment of methodologies and approaches to assessment, sometimes referred to as decision support tools. The criteria are adapted from Corral Quintana [21], Guimarães Pereira *et al. *[22], Van der Sluijs *et al*. [23], and Krayer von Krauss [10]. Main elements being:

1. Fitness for purpose

2. Accessibility

3. Robustness

4. Legitimacy

5. Informativeness

**Figure 1:**
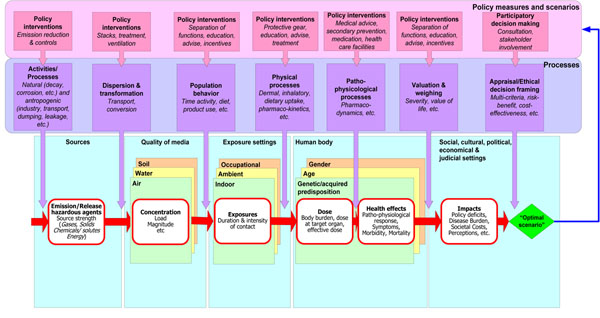
**Towards a practical appraisal framework for complex environmental health problems.** In: Lebret E, Knol A, van Kamp I, Briggs D, Tuomisto J: First draft of Deliverable 9 in INTARESE project; 2007.

However applying such an approach in the case of DEHP with 10 categories to 191 individual articles done by 4 independent topic experts would have resulted in an unbearable workload considering the constraints in project resources and very likely in an excess of bias. Therefore, finally, the following approach for knowledge evaluation was decided upon. First a literature review was conducted in order to map current status of scientific understanding of the topical issues. In parallel for each topical issue a specific causal diagram was developed as to draw a mental picture of the causal chain from exposure to health effects and societal consequences. This approach is based on the general diagram (Fig. 1) from the EU-project INTARESE (http://www.intarese.org/). On the basis of both the literature review and the causal diagram an online evaluation was organized in order to assess the current state of scientific knowledge on the topical issues. A first questionnaire was developed for this, based on the scheme of confidence levels which is also used by the IPCC (Table 1). With respect to most causal elements questions were posed to selected groups of scientists with experience in the relevant field of expertise, based on the confidence scheme. The ambition was to make an inventory of the most pressing knowledge gaps and to be open to potential difference of opinion amongst scientists. This resulted in living up to the expectations of the first ambition mentioned in the beginning of this report, be it mainly from a natural scientific perspective; the societal impacts and aspects were not really addressed at this stage, and the focus was mainly on knowledge gaps, and thus on science itself.

**Table 1:** Scheme to express level of confidence.

4 Very high confidence.	3 High confidence.	2 Medium confidence.	1 Low confidence.	0 Very low confidence.
At least a 9 out of 10 chance of being correct.	At least an 8 out of 10 chance of being correct.	At least a 5 out of 10 chance of being correct.	At least a 2 out of 10 chance of being correct.	Less than a 1 out of 10 chance of being correct.

In: IPCC: Fourth Assessment Report. Climate change 2007: Synthesis report; 2007.

### Second phase: policy interpretation

The second ambition, the relevance for a policy perspective, was reanimated by interdisciplinary dialogue between a social scientist who joined the project at a later instance and the coordinator with a background in assessment of quality of knowledge. The central focus developed here was the question which kind of policy action experts consider to be justifiable based on the identified state of scientific knowledge. This resulted in the development of a second questionnaire regarding policy interpretation and as such adding to the perspective of problem knowledge (phase 1) the meaning of knowledge from a problem solving perspective (Additional file 1). On the basis of the outcomes of the first and the second questionnaire a workshop was organized with a selected group of environment and health experts. The main aim was to bring the results into dialogue and as such to test the outcomes of the analyses of the questionnaires and to enrich the assessment with the interactive and argumentative input of experts. Another issue being discussed in the workshop was the question of what could be done with the outcomes of the project; this will be discussed under the third phase.

### Third phase: reporting and evaluation

Considering the results of the first two phases, it was concluded that some kind of reporting of the outcomes to both policy makers and other stakeholders would be worthwhile. Preliminary reports were drafted based on all results and sent to the experts participating in the workshop for comments. The experts were also asked whether they would like to be acknowledged in any kind of reporting or rather stay anonymous. Moreover they received a short evaluation questionnaire so as to give feedback on the project (Additional file 2). The final format that resulted from the designing and feedback activities with respect to the reporting was a policy brief (Additional file 3). The final part of the process was assessment of whether the end product, the policy briefs, were useful for the target audience. Around forty persons, either policy makers working in the EU bodies, national ministries or pollution agencies, or people working for NGO’s were approached and asked to review the policy briefs on decaBDE, HBCD and phthalates. One single evaluation questionnaire consisting of seven questions was made for all three briefs and persons approached could choose the most relevant brief or review all of them (Additional file 4).

## Results

During the HENVINET project several environment and health topics were dealt with by application of the above described method. Not all topics though were able to go through all phases of the process. In total seven specific topics were addressed: decaBDE, HBCD, phthalates, CPF, nanoparticles, the impacts of climate change on asthma and other respiratory disorders, and the influence of environment health stressors on cancer induction [references topic papers]. The online evaluations of current state of scientific knowledge of all topical issues were finalized. Five workshops were organized based on the outcomes of the knowledge evaluation and the policy interpretation: decaBDE, HBCD, phthalates, CPF and the impacts of climate change on asthma and other respiratory disorders. And finally, as a result of the workshops, four policy briefs were produced [24-27]. We will focus here on the practical methodological experiences from the perspective of experts that were consulted and of some policy experts.

### Evaluation by experts consulted by HENVINET

We present feedback from the experts that participated in the assessment process, be it only those who participated in all phases of the evaluation. This will limit the feedback not only to those experts, being a selection of the broader group that participated in the first online evaluation, but also to experiences in those environment and health issues that were able to realize all steps of the process: decaBDE, HBCD, Phthalates, the impacts of climate change on asthma and other respiratory disorders and the pesticide CPF. Experts who participated in the other environment and health issues thus are not included in the evaluation, as they did not take part in the workshop discussions nor filled out an evaluation questionnaire.

The response rate to the evaluation questionnaire was rather high (Table 2). The amount of time spent on the knowledge assessment questionnaire differs enormously between experts, as well as between topic groups (Table 3). The initial indication used in the beginning that the first questionnaire would only take about 15 minutes thus clearly was an underestimation. Different reasons for input of large amounts of time are mentioned: it was somewhat unclear to some experts the purpose of the assessment and their tasks, and consultation of background information (literature review and sometimes other information as well) with respect to the complicated topics dealt with in both questionnaires demanded extra time.

**Table 2:** The response rate to the evaluation questionnaire

Topic group (*chronological order workshops*)	Number of participating experts in the workshop	Response evaluation
Phthalates	5	4

Brominated flame retardants*	8	8

Impacts of climate change on asthma and other respiratory disorders	8	7

Pesticide CPF	2	3**

*Total*	*23*	*22*

*The group of experts for the brominated flame retardants decaBDE and HBCD was the same; this group received one evaluation questionnaire with respect to both specific brominated flame retardants

**Including the organizing CPF-experts as one respondent, *because of the low rate of participation for the workshop.*

**Table 3:** Time needed to fill out the questionnaires

Topic group	Questionnaire knowledge gaps *range*	Questionnaire policy action *range*
Phthalates	45 minutes – 2 days	1 – 2 hours

Brominated flame retardants*	1 hour – 1 to 2 days	1 – 2 hours

Impacts of climate change on asthma and other respiratory disorders	30 minutes – 4 hours	30 minutes – 2 days

Pesticide CPF	*Not part of the evaluation*	1 – 3 hours

The majority of experts clearly are positive about the extent to which the most important issues were highlighted (Table 4). Only a minority of the experts indicated some important issues that were left out (Table 5). Mostly technical specialized issues were mentioned. An exception was ethical issues: one expert referred to “*The Declaration of Helsinki*” stating that doing harm to humans is not allowed in medical research, whatever the goal of that research might be. This expert proposed to extend this ethical principle to the use of chemicals, e.g. with respect to flame retardants with possible health risks: doing harm to e.g. children by using such chemicals according to this principle might be in conflict with the aim of prevention of harm by fires. In general normative or social issues were hardly mentioned by the groups of experts; they mainly focussed on technical matters in relation to their expertise.

**Table 4:** Did the questionnaires and workshop successfully highlight the most important issues related to the environment and health topics?

Topic group	Positive	Positive/negative	Negative	Do not know
Phthalates	3			1

Brominated flame retardants*	7			1

Impacts of climate change on asthma and other respiratory disorders	6	1		

Pesticide CPF	2	1		

*Total*	*18*	*2*		*2*

**Table 5:** Were any important issues were left out?

Topic group	No	No/Yes	Yes	Do not know
Phthalates	3			1

Brominated flame retardants*	3		4	1

Impacts of climate change on asthma and other respiratory disorders	6		1	

Pesticide CPF	2			1

*Total*	*14*		*5*	*3*

In general most experts are positive, and sometimes very positive about the approach (Table 6). Some mention pros and cons of the approach. The main negative critique to the approach seems to be that it is not sophisticated enough with respect to the complexity of the topics, especially regarding the technical issues. As such, according to several experts, it cannot stand the test of comparison to risk assessment: it is too superficial for this. A related problem is that in the approach being used here, experts were expected to be an expert in all aspects of the complex topics, when in fact they were not, or at least did not always feel at ease with this. Another expert questions whether such approach will indeed come up with new knowledge. Also to some experts lack of clarity about the process and their role was a negative aspect.

**Table 6:** The general impression of the approach

Topic group	Positive	Positive/negative	Negative	Do not know
Phthalates	3	1		

Brominated flame retardants*	3	4	1	

Impacts of climate change on asthma and other respiratory disorders	6	1		

Pesticide CPF	*Not part of the evaluation*			

*Total*	*12*	*6*	*1*	

Positive critique is pointed to the fact that it was an innovative approach that was considered interesting and promising. One expert even considered the approach better than risk assessment as it seems to be more up to date on scientific information than most risk assessment documents. Moreover the combination of experts offered the opportunity to learn from and discuss diversity of interpretation from different perspectives.

Amongst suggestions for improving the approach, more clarity about the approach, of the questions (the level of confidence was mentioned as being unclear) and about the end product, were indicated as important. Also more time was asked for consultation of and discussion about all relevant information. Furthermore a solution was asked for with respect to topics outside the expertise of experts; one suggestion might be to divide the questionnaires in subgroups for different fields of expertise. Diversity of expertise is considered important because of the complexity of the combination of relevant aspects, but is difficult to oversee for individual specialists. Also it was considered important to organize a good balance between different fields of expertise. Transparency about the background of experts is a related issue: it should be clear e.g. if experts have a relation with industry. More in general does any composition of expert panels run the risk of bias because of over- or underrepresentation of specific types of expertise: this is an important issue to consider. With respect to the involvement of experts it was suggested to recruit a large panel so as to ensure that enough will remain even when some drop out.

When asked about the possibility of a stakeholder workshop most experts welcomed the idea (Table 7), even though (to some) certain aspects were unclear. E.g. the question who would be relevant stakeholders was unclear: policymakers and risk assessors were mentioned by one expert, industry by another. One expert pointed out that a balance of views is important. One expert was explicitly negative, stating this will probably lead to ‘prestige-filled confrontations’, thus questioning the relevance.

**Table 7:** Do experts support the idea of involving stakeholders in a final workshop that would consider the contents of the workshop report from a societal perspective?

Topic group	Positive	Positive/negative	Negative	Do not know
Phthalates	3			1

Brominated flame retardants*	5	1		2

Impacts of climate change on asthma and other respiratory disorders	5		1	1

Pesticide CPF	3			

*Total*	*16*	*1*		*2*

The majority of experts indicated they were willing to be acknowledged. As one expert put it: ‘*I am not ashamed to be an expert*’. After (sometimes intense) discussions with the experts about the final text of the policy briefs, some minor shifts could be noticed in favour of acknowledgement (Table 8 and 9). Thus we may assume that the close involvement of experts in the design of the final output may be important for them to gain trust in the end product.

**Table 8:** Do the experts want to be acknowledged in the report on results or do they prefer to stay anonymous? (First indication in evaluation questionnaire)

Topic group	Acknowledged	Anonymous
Phthalates	1	3

Brominated flame retardants*	7	1

Impacts of climate change on asthma and other respiratory disorders	7	-

Pesticide CPF	*Not part of the evaluation*	

*Total*	*15*	*4*

**Table 9:** Actual acknowledgement after final feedback on the policy briefs

Topic group	Acknowledged	Anonymous
Phthalates	3*	3

Brominated flame retardants*	8**	-

*Impacts of climate change on asthma and other respiratory disorders*	*Not yet finalized*, *but all indicated they wanted to be acknowledged*	
	
	*7*	

Pesticide CPF	2	

*Total*	*20*	*3*

*In fact three experts are acknowledged, *including one not taking part in the evaluation. One expert taking part both in the design of the policy brief and the evaluation is not included in the number of workshop participants* (*see above*), *as she was ill at the time of the workshop*

**In fact nine experts are acknowledged, *including one not taking part in the evaluation. One expert taking part both in the design of the policy brief and the evaluation is not included in the number of workshop participants* (*see above*), *as she was ill at the time of the workshop*

Partly the evaluation issues touched upon in the evaluation questionnaire also were discussed in some of the workshops. Without wanting to repeat too much, we will address some of these issues a little bit more in detail as well as introducing some other issues of importance to evaluation of the approach.

#### I don’t know?

Intense discussions were held within the HENVINET consortium about the need to include an ‘I don’t know’-option in the first questionnaire on knowledge gaps. Not using this option would have the benefit that response rates to all questions would probably be higher. Also one may ask if these carefully selected experts are not able to answer these questions, who in fact will be? A disadvantage would be that experts would give answers even though they were not sure about their answer or even merely guessed an answer. An example from one of the participating experts:

“*The phrasing of the questions was weird and made it difficult. E.g. if you have a sediment sample*, *how big is the chance of guessing the correct concentration of deca in that sample*, *or did you mean*, *how great is the chance of finding deca in that sample at all? I ticked off low when didn’t know*, *that’s how I interpreted it. Additional explanations/guidelines would have been of great help. I felt confident answering the environmental matrix questions*, *while in the toxicology part I felt sometimes on thin ice.*”

One may question the value of such answers, and afterwards it is impossible to distinguish knowledgeable from unknowledgeable answers. Another disadvantage might be that experts refuse to fill out the questionnaire as a whole, which may result in even lower response rates. In this case of knowledge evaluation it was decided not to use the “*I don’t know*”-option in the first questionnaire. Like a boomerang this returned on the discussion table during some of the workshops in which several experts pointed out a feel of discomfort of not being able to indicate lack of expertise on issues when in fact this was the case. One of the organizers of the workshops responded to this issue by pointing out the importance of the step by step character of the approach and of the workshop discussion:

“*The goal is not to produce and publish these results without the discussion we will have here today. Our aim is to produce a policy advice. We therefore wanted your answers to all questions even if it was not your core area of expertise and have by purpose not included an “opt-out” option in the questionnaire. The reason we are here is to focus on the discussion around these issues. The results from the first questionnaire are meant to provoke your thoughts and to channel a discussion.*”

#### Interpretation bias

An issue related to the previous one is that in general it was considered questionable if the understanding of the questions by the diversity of experts was similar and coherent, both between different sub-disciplines as well as within sub-disciplines. The questions leave room for diversity of understanding which could cause difference in interpretation that would perhaps not have occurred when questions were understood similarly. This may cause a bias in the response. We give two examples:

Example 1: “*Just as an example*, *I was just looking at how I answered the very first one: human epidemiological studies*, *adverse effects of HBCD*, *and the question is: “Based on human epidemiological studies what is your level of confidence in the scientists’ ability to predict adverse effects of HBCD in males and females”*, *and I put very low because as far as I know there aren’t any epidemiological studies of HBCD in humans to base any kind of prediction on*, *so I put very low*, *because I don’t know*, *because no-one else knows*, *because there is no data in the literature to tell me whether or not that’s the case. I could try to extrapolate from animal studies but that’s not what it is asking*, *it’s asking me for my confidence on epidemiological studies that don’t exist. So that’s how I interpreted some of these questions*, *that if there is no data then you can’t put that I have medium confidence*, *I don’t know what HBCD is going to be doing in humans because nobody has done the studies yet. And I don’t want to try and predict it*, *because that is not science that should be in a crystal ball.*”

Example 2:“*Just a comment to the questions. When I was filling in this I was often in the situation that I knew about a few studies that I believed in*, *but I still think that there is too little data. So where do I put my checkmark? I believe the data but I think they are too few*, *so maybe I ended up in the middle*, *and maybe someone else considered the same situation differently? Just a comment.*”

It was suggested that the phrasing of the questions could be clearer and that also better information about the purpose and meaning of the evaluation and the questions should be given.

#### Other potential biases

Several other potential biases were mentioned in the workshop discussions. In the workshop on climate change the *geographical background* of experts was noticed as a bias. The issue of dampness was considered as one of the important issues in the causal diagram. The workshop participants discussed whether this was caused by the main role of Scandinavian experts in the development of the causal diagram as experts from southern Europe are confronted much less with this issue due to higher temperatures in their region. Similarly, increasing exposure to house dust mites may be a consequence of climate change but only in Northern Europe.

In the same workshop another potential bias was discussed: many of the workshop participants were already familiar with members from the HENVINET project on a personal level. This clearly appeared to be an incentive to participate. Thus we can conclude that *personal relations between experts* potentially carries the risk of expert bias. In general many of the experts were not very keen on answering questionnaires from various kinds of web portals like the HENVINET or from e.g. EU-polls. As one expert puts it:

“*I replied to the questionnaire as a friend of ...* (*name of one of the organizing HENVINET experts*), *otherwise I had never replied.* (*...*) *To be honest*, *I don’t like this type of interview*, *i.e. in certain web portals. Most experts are not keen in answering this type of questionnaires.*”

One way to avoid this kind of non-response might be the use of a personal interview which is possibly also more informative than a questionnaire. Also a small fee may convince a broader group of experts to participate.

An *imbalance of types of expertise* within a group of experts may cause a bias. With respect to the group discussion *dominance in the discussion of one or several participants* might be another bias. Also the possible *linkages of experts to certain stakes*, like industry or other social organizations, may cause a bias. Independence, a quality that several of the HENVINET-experts considered to be of main importance as a selection criterion for participating experts, is to be questioned per definition according one expert:

“*All EU advisers are dependent on funding*, *so there might be a bias.*”

Transparency on the selection of experts and on expert profiles is considered as one remedy for such bias not being taken into account when judging the outcomes.

#### Einstein and the need for (not too much) simplification

With respect to quality of the endeavour even Einstein was mentioned in the discussion, referring to the quote “*Everything should be made as simple as possible*, *but not one bit simpler.*”, in this case freely translated into “*Simplify as much as possible*, *but not further*”. The important issue raised here is how to balance ambition, complexity, urgency, pragmatics and quality. The ambition of embracing the full causal chain in this project maybe praiseworthy considering the combination of complexity and importance of public health risk, but runs the risk of becoming too superficial due to lack of information, lack of in depth review and lack of relevant expertise. This may put pressure on the quality of the assessment and outcome. As such, some experts questioned the quality of the assessment. On simplification some members of the HENVINET project team responded by acknowledging this to be an important issue, while simultaneously pointing out that pragmatic choices also had to be made in order to keep the project manageable; two examples:

“*We started off with about ten different criteria*, *each with its own scale. For example amount of empirical data would have been one of those criteria. So you say*, *well*, *the method that was used was very good*, *except that it has only been used once or twice*, *so we need more. But the problem is that it just completely overwhelmed both the experts and us* (*…*) *it was unmanageable.*”

**“***We counted it once and I think we come up with 290 parameters that you would have to judge for each of the questions.*”

Some experts indicated that the use of a thorough literature review as a basis for assessment for all participating experts might be a good solution. Still, experience in the workshops shows that even then, there will remain enough room for discussion; one example regarding limited availability of studies on specific aspects:

One expert: “*I did a pubmed search on decaBDE and only got 5-6 pure toxicology studies among all the 50-60 hits*, *I concluded there is little knowledge on this and think it is odd that some evaluators have ticked high or even very high confidence on certain toxicology questions.*“

Another expert responding: “*There is no reason not to have faith in the few studies there are. How much documentation do you need? There are some publications on neurotoxicity*, *and I also trust the results reported by NTP in 1987. But of course there is still more to be done. This is an interpretation issue.*”

#### Guinea pigs, self-confidence and a sense of urgency

“*Cannot agree that this is a group of experts*, *we were selected as guinea pigs*, *but not as a risk assessment group. You must make a distinction on how far you can go. I do not feel comfortable in serving policy makers conclusions.*”

This statement by one of the experts exemplifies what was said in the previous section on the quality of the scientific basis for the assessment. Still, not all experts responded in the same way. Some were more self-confident in being able to give policy advice, and in fact in the end the majority of experts felt confident enough to be acknowledged in the policy briefs that were the output of the project. Even the expert being quoted above, after intense consultation on the content of the policy brief, changed position from not wanting to be acknowledged to wanting to be acknowledged. This does not exclude however the possibility that experts may still remain divided on the issue of weight of knowledge. On the one hand this brings us back to questions on the validity of the evaluation from a quality perspective as well as issues of experts’ self-confidence, and, on the other hand the important second ambition of the HENVINET project: facilitating the use of knowledge in a policy context. Here a sense of urgency may be of importance considering the potential public health risks, as one of the scientists involved in the HENVINET project stated:

“*I met …* (*name deleted for anonymity*) *once in a local meeting*, *the one involved in endocrine disruption in fish in England*, *and he was the driving force behind the initial ban in organic tin compounds*, *he said it took 20 years from the first time we found negative effects to the ban. And we all grew up with the DDT story*, *the PCB story*, *and the Tributyltin* (*TBT*) *story*, *and maybe now also with the PBDE story*, *and maybe now on the first or second day of the HBCD story. And every time we see a compound that is doing more or less the same and has the same properties*, *I think at some stage we should be proactive.*”

This brings us to the weight of current, and often limited, scientific knowledge for policy action. Clearly this cannot be objectified based on (the review of) scientific findings: ambiguity will potentially always be part of the game. As one expert stated:

“*An expert is never objective. The science is not objective. It is important to know potential bias.*”

The diversity of opinion within some of the expert groups consulted in this project regarding weight of knowledge for restrictive policy action, e.g. a ban on the use of a specific compound, exemplifies this. An important related issue raised in one of the workshops is the responsibility of scientists; as one expert put it:

“*It is important to identify the role we have as academic scientists when it comes to policy actions. There is a misuse of scientists. We work towards NGOs*, *politicians and industry. Then*, *we are not always acting as scientist. We could agree on some things on one side*, *but policy makers have to make their own conclusions. Scientists have limited impact on policy making. Clear data are needed. Role definition is important and we should not mix up functions. HENVINET is on the way in this context.*”

Someone then asked: “*Should we ask scientists about science only and not about policy actions?*”

The expert responded: “*You will get into trouble if you suggest something like management*, *how to solve the problem. We need to have an independent group which is pure science based now when industry is moving forward in a rather aggressive way.*”

Someone else asks: “*So*, *what you say is that solving the problem is not our responsibility? There might be technical problems or other issues in this that is not for everyone to understand and then experts are needed.*”

The expert responds: “*We do have a responsibility*, *for example to find out what we agree on. Industry is working on its own; scientists are working on their own. We have to be on both arenas*, *but we must know when we are doing what*, *the roles are not well defined.*”

How precisely this can be arranged in a good manner remains unclear, even though most participating experts in general are positive about the approach developed in HENVINET.

#### Broader horizon, diversity and interaction bonus

In particular the opportunity to widen one’s own horizon and to interactively exchange knowledge and debate with a diversity of experts seemed to be well appreciated in this approach. Different parts of the approach also helped in focussing on specific relevant aspects of scientific knowledge, and as such can be considered of reflective value. With respect to the use of the causal diagram one expert described the bonus as follows:

“*The scheme compels you to focus and to broaden your perspective and to see where I have knowledge.*”

Another expert described the benefits of the whole sequence of steps in the process as follows:

“*I must say that the first questionnaire was difficult*, *but it was interesting to see how much I felt like I knew and how much I felt like I didn’t know. But I found the second questionnaire quite thought provoking*, *because it suddenly dawned on me why I was answering the first questionnaire. And suddenly the policy and things like that. Because then when you ask why*, *you have to motivate why do I tick this box. I think that was a good exercise to sort of make me formulate things. It was good to have had those two steps before coming here*, *because otherwise we would have sat here talking about the science*, *and not about what does it all mean? In that sense I think it was a good exercise to do it twice. Once to start with*, *and then get the results*, *look at it and then do the next step. So I think that was very good actually*, *so then today could be very focused.*”

In comparison with risk assessment some stated this approach to be of complementary benefit:

“*Reports after risk assessments often take long time to write and may not reflect the latest data. We should not put this group aside. This is an intermediate stage. You will get different answers depending on who you ask; public*, *scientists*, *risk assessors.*”

And one expert responded to the question whether the approach is worthwhile: “*You won’t get that answer until you go back and see if people with a policy level are willing to listen to what this panel says. Because if they say: We have a risk assessment*, *why should we listen to this group? Then it didn’t have any effect. I don’t think that we can influence that outcome. It’s what happens at the next step and how that is taken. Because this is a problem with a lot of risk assessment now that they don’t like having new science brought in when they think that they are almost done. It muddies the water. Especially if it raises issues that they thought weren’t a problem. But if they find that this is valuable way of complementing a risk assessment*, *then I think you have done a good job. Then I think it is a very valuable way of doing it.*”

### Evaluation by policy makers

Only three policy makers reflected on policy briefs. Bad timing, too long an e-mail introducing the project and lack of time may be the reasons for the poor response rate. The questionnaire was also sent close to the end of the project, leaving little time for approaching more people or promoting it further. One of the three respondents commented on the content without using the questionnaire and criticized the way it was sometimes presented:

“*The document is not very much supportive for the WHO policy on encouraging breastfeeding*, *but it is not clear either from the document that artificial powders are less contaminated. I would suggest to be more prudent on the issue of mother’s milk. It is very sensitive*.”

It was also commented that when discussing a ban, the brief was very black and white in that a ban seemed to be the only regulatory option to reduce exposure. This was confusing according to the policy maker.

On the question as to whether the approach would yield information useful to policy making, the two experts answering the questionnaire were fairly positive, although more in depth analyses would be necessary and much of the information could be found elsewhere:

*“The ‘policy brief reports’ are concise and easy-reference. This may be helpful for policy makers. However*, *I do not think they are essential for the decision making*, *because the information is available anyway.”*

Both respondents to the questionnaire answered yes to the question on whether the causal diagram was easy to understand and useful to policy makers.

To the question on how and to which extent the expert opinions could be used in the policy process, it was again mentioned that it is a good starting point for more in depth analyses. Another policy maker stated that the briefs seemed to be of most use to research policy, while a third meant that they could have a broader use:

*“International and national organizations*, *such as EC*, *ECHA*, *EPA*, *… publish risk assessments and overview documents. In policy making we will make use in the first place of the information in such documents. The HENVINET brief reports are useful because an overview is given of the published* (*and to be published*) *documents*.”

None of the policy makers suggested any improvements to the procedure or format to make the briefs more useful to policy makers; however, one respondent had additional advice:

*“Make link with existing ‘good practise’ policy actions on the different topics* (*including e.g. awareness raising*) *which can be put in to practise by regional or national governments in combination with the policy instruments at EU level”*

## Conclusions

The methodological development of the HENVINET knowledge assessment approach proved to be largely topic and context specific: depending on the complexity of the topics under discussion and depending on the context of actors involved in the process, ambitions will have to be moulded accordingly. And perfection, if this could be defined, seemed out of reach all the time. Nevertheless, many involved were quite positive about it. It seems that many felt that it fits some important needs in current science when addressing the needs of policy making on such important issues, without anyone really having a clue on how to actually do this.

Some rather fundamental issues occurred during the process, challenging the experts involved, both from the HENVINET project as well as those participating in the process. Challenging questions remain on the quality of such approach and its product. No objective, unambiguous or perfect criteria appeared within our reach to decide e.g. on the status of scientific knowledge: when do ‘we’ know enough for what and who decides? What is the meaning and weight of knowledge? How do we decide what is the relevant body of knowledge or the ‘right’ (group of) experts? Does the fact that we cannot easily define or objectify this mean that anything goes and that it does not matter who decides on what? Practice tells us that there probably is no best method and that the best we can do is dependent on contextual negotiation and learning from experiences that we think are relevant.

The HENVINET approach contained several innovations with respect to mainstream practice of most environment and health experts involved in this project. In the assessment of the state of the art of science the approach, perhaps the use of literature review and of causal diagrams was not new, but in combination with the use of confidence levels and as such the use of qualitative assessment, it was quite new in this field of practice. Also openness to potential difference of opinion between experts is not very common in this field of practice. In the assessment of weight of current knowledge for policy making the introduction of the problem solving perspective seemed quite uncommon. The interdisciplinary cooperation in parallel posed quite some challenges and to some extent sometimes can be described as a clash of cultures. The transdisciplinary challenge (involvement of non-scientific actors), originally part of the projects’ ambition was not realized due to lack of time and resources. The involvement of policy representatives and stakeholders would perhaps have created a different and more policy relevant dynamic. Still, efforts were made that will ease steps in this direction.

## Competing interests

The authors declare no competing interests.

## Authors’ contributions

HK was responsible for the methods development and assessment, and has written the article. MKK co-designed the classification schemes and the methodology, and led the work. ACG, KEZ, SR, ER, GSE, MS, BM and BF were responsible for design, implementation and analysis of results of the case studies. AY co-designed and managed the web applications, contributed to the data collection, implementation and analysis. AB managed the project, and contributed with discussion, analysis and expertise to the knowledge evaluation methodologies and results interpretation. All authors reviewed the article and approved the final version. MS edited the English.

## Supplementary Material

Additional file 1 Focus of the second questionnaire Click here for file

Additional file 2 Focus of the evaluation questionnaire Click here for file

Additional file 3 Focus of the policy brief Click here for file

Additional file 4 Major questions asked in the policy brief evaluation Click here for file
