# Supplementary material for: We’re only in it for the knowledge? A problem solving turn in environment and health expert elicitation
Source: Environ Health. 2012 Jun 28;11(Suppl 1):S3. doi: 10.1186/1476-069X-11-S1-S3 (PMC3388440; doi:10.1186/1476-069X-11-S1-S3)
Supplement: Additional file 1 — Focus of the second questionnaire [file 1476-069X-11-S1-S3-S1.pdf]

Additional file 1 – Focus of the second questionnaire

1. Prioritization of the most important elements of the causal diagram according to their influence on the extent of the health risk the causal chain leads to.
2. The type of action experts consider to be justified by the evidence available; this can range from fundamental research to applied research with respect to scientific action, and from monitoring and awareness raising to restrictive or prohibiting activities with respect to policy making.
3. The level of confidence that conducting more scientific research would yield decisive knowledge within the next five years.
4. The level of confidence in the possibility that policy actions to effectively manage this health risk will become technically (not politically) feasible within the next five years.
5. The extent to which experts think the current scientific knowledge of the overall problem represents sufficient evidence to justify policy measures (e.g. a ban or similar restrictive action with respect to toxic compounds) to decrease the health risk.
