# Supplementary material for: We’re only in it for the knowledge? A problem solving turn in environment and health expert elicitation
Source: Environ Health. 2012 Jun 28;11(Suppl 1):S3. doi: 10.1186/1476-069X-11-S1-S3 (PMC3388440; doi:10.1186/1476-069X-11-S1-S3)
Supplement: Additional file 2 — Focus of the evaluation questionnaire [file 1476-069X-11-S1-S3-S2.pdf]

Additional file 2 – Focus of the evaluation questionnaire

1. Time needed to fill out both questionnaires
2. Whether the questionnaires and workshop successfully highlighted the most important issues related to the risks of the environment and health topics
3. Whether any important issues were left out
4. The general impression of the approach
5. Suggestions as to how the approach could be approved
6. Whether experts support the idea of involving stakeholders in a final workshop that would consider the contents of the workshop report from a societal perspective
7. Space for remarks
8. Do the experts want to be acknowledged in the report on results or do they prefer to stay anonymous
