# Supplementary material for: We’re only in it for the knowledge? A problem solving turn in environment and health expert elicitation
Source: Environ Health. 2012 Jun 28;11(Suppl 1):S3. doi: 10.1186/1476-069X-11-S1-S3 (PMC3388440; doi:10.1186/1476-069X-11-S1-S3)
Supplement: Additional file 3 — Focus of the policy brief [file 1476-069X-11-S1-S3-S3.pdf]

Additional file 3 – Focus of the policy brief

Key Messages

Policy context: Key information on the specific environment and health issue

Policy options: Key issues based on the answers from both questionnaires and discussion at the workshop

Executive summary

Situation: Information on the specific environment and health issue with respect to e.g. the purpose of use of a chemical, policy measures with respect to the production and use of the chemical and the response of producers.

Background: State of the art scientific knowledge based on the literature review, the causal diagram and a sketch of the steps in the assessment process

Assessment: Results of the assessment: priority knowledge gaps and policy considerations

Recommendations: Key messages from the assessment

Acknowledgements: Naming those experts that were willing to be acknowledged

Authors, contributors, contact information and information on the HENVINET project

References
