# Supplementary material for: We’re only in it for the knowledge? A problem solving turn in environment and health expert elicitation
Source: Environ Health. 2012 Jun 28;11(Suppl 1):S3. doi: 10.1186/1476-069X-11-S1-S3 (PMC3388440; doi:10.1186/1476-069X-11-S1-S3)
Supplement: Additional file 4 — Major questions asked in the policy brief evaluation [file 1476-069X-11-S1-S3-S4.pdf]

Additional file 4 – Major questions asked in the policy brief evaluation

1. Do you think the HENVINET approach of expert elicitation process will yield useful information to help decision making (please indicate why)?
2. Do you find causal diagram easy to understand and a helpful format to inform policy makers and stakeholders about health risk issues and identify priority knowledge gaps for endocrine disrupting compounds?
3. To what extent and how would you use the expert opinions provided, regarding the extent to which current scientific knowledge justifies a policy intervention elicitation, as a useful contribution to the policy process?
4. Do you think the procedure and/or format should be improved to be used by policy makers working on endocrine disrupting issues? How could they be made more useful?
5. Do you have additional advice for us?
